# Microbiota Characterization of Compost Using Omics Approaches Opens New Perspectives for Phytophthora Root Rot Control

**DOI:** 10.1371/journal.pone.0158048

**Published:** 2016-08-04

**Authors:** Josefa Blaya, Frutos C. Marhuenda, Jose A. Pascual, Margarita Ros

**Affiliations:** 1 Department of Soil and Water Conservation and Organic Wastes Management, Centro de Edafología y Biología Aplicada del Segura (CEBAS-CSIC), Murcia, Spain; 2 Department of Agrochemistry and Biochemistry. University of Alicante, Alicante, Spain; Universita degli Studi di Pisa, ITALY

## Abstract

Phytophthora root rot caused by *Phytophthora nicotianae* is an economically important disease in pepper crops. The use of suppressive composts is a low environmental impact method for its control. Although attempts have been made to reveal the relationship between microbiota and compost suppressiveness, little is known about the microorganisms associated with disease suppression. Here, an Ion Torrent platform was used to assess the microbial composition of composts made of different agro-industrial waste and with different levels of suppressiveness against *P*. *nicotianae*. Both bacterial and fungal populations responded differently depending on the chemical heterogeneity of materials used during the composting process. High proportions (67–75%) of vineyard pruning waste were used in the most suppressive composts, COM-A and COM-B. This material may have promoted the presence of higher relative abundance of Ascomycota as well as higher microbial activity, which have proved to be essential for controlling the disease. Although no unique fungi or bacteria have been detected in neither suppressive nor conducive composts, relatively high abundance of *Fusarium* and *Zopfiella* were found in compost COM-B and COM-A, respectively. To the best of our knowledge, this is the first work that studies compost metabolome. Surprisingly, composts and peat clustered together in principal component analysis of the metabolic data according to their levels of suppressiveness achieved. This study demonstrated the need for combining the information provided by different techniques, including metagenomics and metametabolomics, to better understand the ability of compost to control plant diseases.

## Introduction

*Phytophthora nicotianae* van Breda de Haan (= *Phytophthora parasitica* Dastur (1896)) stands out among plant pathogens since it is a threat to plant productivity on a global scale for a broad range of hosts [1]. The host range of *P*. *nicotianae* includes 255 plant genera in 90 families [2]. In Spain and Tunisia, *P*. *nicotianae* causes root- and collar- rot in pepper plants (*Capsicum annuum* L.) and has become a major disease during the last years [3–5]. Management of this disease is based on soil fumigation using compounds such as methyl bromide, 1,3-dichloropropene, chloropicrin, metalaxyl and mefenoxam [6]. The banned use of most of these products and their inability to totally control the disease, have prompted the exploration and identification of new approaches.

The use of compost made of agro-industrial waste and by-products is a promising alternative. These composts are not only free of xenobiotics and excessive content of heavy metals but also, they have been proven to suppress a wide variety of soil-borne plant pathogens [7–9]. Suppressive composts are examples of natural biological control of diseases as the result of a three-way interaction between the microorganisms in the compost (composition, diversity and function), plant pathogen, and plant [10]. The extant microbiota of composts has shown to be the main factor responsible for suppressiveness [11,12]. In particular, suppressive effects against *Phytophthora* spp. have been related to the ability of composts to maintain a high microbial activity [7]. Nevertheless, the mechanisms of suppression of these oomycetes have not been unraveled.

Compost microbiota highly depends on the materials used during the composting process. The quality of compost organic matter is important with respect to the efficacy of the suppression and the regulation and maintenance of microbial communities in composts [13]. Shifts in the chemical composition of compost organic matter have been previously characterized by ^13^C- Nuclear Magnetic Resonance (^13^C-NMR) spectroscopy [14]. However, its ecological significance should be elucidated in relation to the microbial communities inhabiting the organic matter [15], which can be studied through molecular analysis—such as fingerprinting techniques and sequencing methods [16–18]. High-throughput sequencing (e.g. Illumina or Ion Torrent) is a powerful alternative for the identification at a greater depth of the microbial community composition and diversity. Among the studies regarding the metagenomics of compost [19–22], only Yu et al. [22] investigated the connections between the microbial communities and the disease suppression ability of compost against *Pythium ultimum*.

It should be noted that genomic information itself is not enough for understanding the biological processes that take place within compost. In the attempt to fully describe the compost microbiome, metabolomics have emerged as a functional approach that provides insights into the metabolic activities engaged by whole communities of microorganisms [23]. Several studies pointed out the central role played by metabolites in cellular activities and the mileage that could be gained by monitoring at the level of the metabolome [24]. Metabolites (low-molecular-weight compounds such as amino acids, sugars, and lipids) play significant roles in the microbial regulation of the central and secondary metabolism. Not only can they contribute to external signals as indicators of the environmental conditions or by sensing such signals, but also they vary in response to a variety of stimuli (e.g. nutritional deficit, external stressors, or disease) [25]. Currently, the implementation of metabolomics for environmental monitoring is still at an early stage, mostly applied as a screening tool to assess the potential toxic effect of pollutants [26].

We hypothesize that suppressive composts will contain more similar microbial communities among them compared to non-suppressive composts, and will depend on the quantity and state of the organic matter in each compost. Based on this, the specific objectives of this study were (i) to study the *in vivo* ability of four agro-industrial waste-based composts to control *P*. *nicotianae* in pepper plants, (ii) to compare the chemical heterogeneity of organic matter present in the different composts, and (iii) to characterize and compare the microbial composition and metametabolome of suppressive and non-suppressive composts.

## Methods

### The composts assayed and their analysis

Four agro-industrial composts were made from different wastes (expressed as dry weight) as follows: Compost A (COM-A): pepper sludge (125 g kg^-1^), pepper wastes (125 g kg^-1^), and vineyard pruning wastes (750 g kg^-1^); Compost B (COM-B): pepper wastes (160 g kg^-1^), artichoke wastes (160 g kg^-1^), and vineyard pruning wastes (680 g kg^-1^); Compost C (COM-C): pepper sludge (190 g kg^-1^), pepper wastes (20 g kg^-1^), garlic wastes (20 g kg^-1^), carrot wastes (350 g kg^-1^), almond shells (40 g kg^-1^), and vineyard pruning wastes (380 g kg^-1^); Compost D (COM-D): artichoke sludge (150 g kg^-1^), artichoke wastes (264 g kg^-1^), vineyard pruning wastes (500 g kg^-1^), and compost (86 g kg^-1^).

The composts were produced in open-air piles of 200 kg, the bio-oxidative phase lasting 75 days and maturation 42 days. The moisture content was initially set at 40–50% and was maintained by watering. The piles were turned periodically to ensure aeration, and the temperature evolution was monitored periodically (data not shown). Once the composting process was finished, the composts were milled and passed through a 1-cm sieve. Three samples of each compost pile were taken by mixing nine sub-samples from random sites within each pile. The samples were stored at -20°C and 4°C for subsequent analysis.

Different physical-chemical, chemical, and biological characteristics of the composts and peat were measured. The pH and electrical conductivity (EC) of the composts and peat were measured in a 1:10 (w/v) water-soluble extract, in a conductivity meter and pH meter, respectively. The total organic carbon (TOC) and nitrogen were measured with an Elemental Analyzer (LECO TruSpec C/N) and nutrients by ICP-OES (ICAP 6500 DUO). Dehydrogenase activity was measured by the method of Garcia et al. [27].

### Organic matter analysis by ^13^C-NMR

The organic matter composition of the four composts and one peat was estimated by spectral intensity integration over regions with chemical shift characteristics of different organic carbon functional groups. The CPMAS ^13^C-NMR experiments were performed in a Bruker Advance DRX500, operating at 125.75 MHz for ^13^C. The samples were packed into a 4–mm-diameter cylindrical zirconia rotor with Kel-F end-caps and spun at 10000 ± 100 Hz. A conventional CPMAS pulse sequence [28] was used, with a 1.0-ms contact time. Between 2000 and 5000 scans were accumulated, with a pulse delay of 1.5 s. The line broadening was adjusted to 50 Hz. Spectral distributions (the distribution of total signal intensity among various chemical shift ranges) were calculated by integrating the signal intensity, expressed as a percentage, in five chemical shift regions: 0–45 (aliphatic structures), 45–60 (methoxy groups), 60–110 (polysaccharides structures region), 110–160 (aromatic structures), and 160–210 (carboxyl, carbonyl, amide C) [29]. The alkyl/*O*-alkyl ratio was also calculated [14].

### DNA extraction, sequencing, and analysis

Total DNA was extracted using the FastDNA^®^ Spin Kit for soil (Q-Biogene, Carlsbad, CA, USA), following the manufacturer´s instructions. The DNA concentrations of the samples were determined using a NanoDrop^®^ ND-1000 Spectrophotometer (Thermo Fisher Scientific Inc., DE, USA); the samples were then stored at -20°C until required. For the molecular analysis of bacterial communities, the 16S rRNA gene was amplified using primer pairs 8F/120R, F388/R534, F968/R1073 and 8F/R361 [30, 31] and for the fungal community, the ITS1 and ITS2 regions of the fungal rRNA gene were amplified using the ITS5/ITS2 and ITS3/ITS4 primer pairs [32]. Each sample was amplified in triplicate; the amplicons were purified using the QIAquick PCR Purification Kit (Qiagen, Hilden, Germany) and composited together at equimolar concentration prior to sequencing. For PCR amplification, each 25-μL PCR mix contained the following reagents: 1X KAPA2G Fast HotStart ReadyMix2 (2X) (Kapa Biosystems, Boston, MA, USA), 1.5 mM MgCl_2,_ 0.5 μM of each primer, and 5 μL of DNA.

The PCR with primers 8F/120R, F968/R1073 and 8F/R361 was performed using the following conditions as follows: 15 cycles of denaturation at 90°C for 30 s, amplification with a temperature gradient of 70°C−50°C for 30 s, and a final extension of 72°C for 30 s. Additionally, samples were held for 30 cycles of denaturation at 94°C for 45 s, amplification at 50°C for 45 s, and a final extension of 72°C for 45 s. The PCRs for primer pair F388/R534 and the ITS region had an initial denaturation step at 95°C for 3 min, followed by 25–40 cycles of denaturation at 95°C for 15 s, amplification at 60°C for 15 s, extension at 72°C for 15 s, and a final extension of 72°C for 1 min.

A library was created using the Ion Plus Fragment Library Kit, and barcodes were added by the Ion Xpress^™^ Barcode Adapters 1–96 Kit. The template preparation was performed with the Ion OneTouch^™^ 2 System and the Ion PGM^™^ Template Kit OT2 400. Finally, the platform sequenced the samples using Ion Torrent PGM (Life Technologies, Carlsbad, CA, USA) with the Sequencing Kit Ion PGM 400, in chips Ion 318 Chip kit and Ion 314 Chip kit.

The data analysis was performed using the software packages QIIME v1.8.0. and USEARCH v7.0.1090. Sequences shorter than 60 bp and/or Q mean quality scores below 25 were removed. Primers and barcodes were removed and a chimera filter was used. The remaining high quality sequences were grouped in operational taxonomic units (OTUs), following the Open Reference method: sequences were clustered against the GreenGenes v13_8, for the bacterial community, and against UNITE/QIIME 12_11 ITS, for the fungal community, using the *unclust* method with 97% similarity. Sequences not matching the database were subsequently clustered *de novo*. A representative set of OTUs was generated and then the taxonomy of each of the OTUs was assigned using the same database. The sequences have been deposited in NCBI under BioProject PRJNA283180.

Shannon diversity was used to estimate diversity of bacterial and fungal communities as indicated by Neher et al. [19]. Shannon diversity was calculated as H´ = -Σ(*pi* ln *pi*) where *p* represents the proportion of taxon *i* in the community.

### Metabolite extraction and analysis

Metabolite extraction was performed by water extraction (1:10 w/v, compost to deionized water) of six replicates of the composts and peat. The mixtures were shaken for two hours, after which the supernatant was passed through a 0.2-μm filter. The supernatant was analyzed using an Agilent 1290 Infinity UPLC system coupled to a 6550 Accurate Mass quadrupole TOF mass spectrometer (Agilent Technologies, Waldbronn, Germany) using an electrospray interface with jet stream technology. Separation was achieved on a reverse phase Poroshell 120 EC-C18 column (3X100 mm, 2.7 μm: Agilent) operating at 30°C. The mobile phases were water:formic acid (99.9:0.1 v/v; phase A) and acetonitrile:formic acid (99.9:0.1 v/v; phase B). An isocratic flow of 95% phase A and 5% phase B was maintained for 3 min. The flow rate was set constant at 0.4 mL/min and the injection volume was 3 μL. The optimal conditions of the electrospray interface were as follows: gas temperature 280°C, drying gas 9 L/min, nebulizer 45 psi, sheath gas temperature 400°C, sheath gas flow 12 L/min. Spectra were acquired in single MS mode with an m/z range of 100–1100, negative polarity, and an acquisition rate of 1.5 spectra/s. Internal mass calibration, by simultaneous acquisition of reference ions and mass drift compensation, was used to obtain low mass errors. Data were processed using the Mass Hunter Qualitative Analysis Software (version B.06.00, Agilent Technologies). After analysis of the data obtained from the metabolic, a peak grouping was carried out, following a script, by R software.

### Suppressiveness bioassay

The pathogenic strain CC2 of *P*. *nicotianae* (accession number KJ000327) previously isolated from pepper plants with disease symptoms was used in this study [4]. The inoculum of *P*. *nicotianae* was produced by transferring one agar plug (5 mm) of 7-day-old mycelia on pea agar medium (100 g L^-1^ ground peas, 100 mg L^-1^ β-sitosterol, and 20 g L^-1^ technical agar, adjusted to pH 5.5), autoclaved at 121°C for 20 min and amended with 100 mg L^-1^ sterilized streptomycin. The culture was maintained at 28°C for 7 days. The mycelia were recovered from the content of two Petri dishes and mixed with 100 mL of sterile distilled water, using a blender.

Composts were mixed with a commercial peat (50/50 v/v) to obtain different treatments: TCOM-A, TCOM-B, TCOM-C, TCOM-D, and TPeat (100% peat, as a control). Seeds of pepper (*Capsicum annuum* cv. Lamuyo) were sown in trays of 150 pots, with one seed per pot and a covering of vermiculite. Six replicates of each treatment were established randomly, each replicate consisting of 10 seeds. Germination was carried out in a germination chamber at 28 ± 1°C. Once the seeds had germinated, the trays were placed in a growth chamber under daylight conditions. Four replicates of each treatment were inoculated with 2 mL of *P*. *nicotianae* (~10^3^ cfu g^-1^ substrate) after the first true leaf appeared. The suppressive effect of the different treatments was determined by measuring the disease incidence (number of diseased plants) 23 days after inoculation.

### Statistical analysis

The physical, physical-chemical, and biological characteristics of the composts and peat, as well as the results from the suppressiveness bioassay, were subjected to one-way analysis of variance (ANOVA). When the F-statistic was significant, Tukey’s post hoc test (*p*≤ 0.05) was used to separate means. Pearson correlations were made between all data. The statistical analyses were performed using SPSS 19.0 software (SPSS Inc., Chicago, IL, USA).

Statistical analysis of metabolite data was carried out with Metaboanalysis 2.5 software. A multi-variant analysis of mass compounds by principal component analysis was used. For heatmap clustering of samples and mass compounds, the squared Euclidean distance and ward linkage were utilized.

## Results

### Physical, physical-chemical, and biological analyses

The main physical-chemical and biological characteristics of the composts and peat are shown in Table 1. Both pH and EC showed significant differences depending on the substrate (F = 745; p<0.001; F = 236; p<0.001, respectively).

**Table 1:** Physical-chemical and biological properties of composts and peat.

	COM-A	COM-B	COM-C	COM-D	Peat
pH	8.5 c	8.9d	8.8d	6.2b	5.5a
EC^a^ (mS cm^-1^)	1.9ab	2.6c	1.8a	3.8d	2.0b
Total organic C (g kg^-1^)	433d	316b	374c	273a	480e
Total N (g kg^-1^)	25d	22b	29e	24c	13a
P (g kg^-1^)	3.7b	4.0c	4.0c	5.6d	0.3a
K (g kg^-1^)	24d	28e	18c	16.6b	0.6a
Dehydrogenase activity (mg INT g^-1^)	27.2d	37.0e	9.4c	3.2b	0.32a

^a^EC, electrical conductivity.

Data are mean of three replicates. For each parameter, data followed by the same letter are not significantly different according to Tukey’s post hoc test (*p*≤0.05).

Peat had the lowest pH values whereas, COM-B, COM-C, showed the highest pH values. Compost COM-A, COM-C had the lowest EC value and COM-D the highest.

The TOC of composts and peat ranged from 273 to 480 g kg^-1^, with the highest values for peat followed by COM-A, COM-C, COM-B and COM-D (F = 3118; *p*<0.001).

As was expected, peat showed the significantly lowest level of values for N, P and K compared with composts. The content of N was in the range 22–28.5 g kg^-1^for composts, P range was 3.7–5.6 g kg^-1^ and K between 16.6–28 g kg^-1^.

Dehydrogenase activity differed significantly among different composts and peat (F = 139; *p*<0.001), composts COM-A and COM-B showing the highest levels and peat the lowest (Table 1).

### The suppressive effect of different growing media

**Fig 1:**
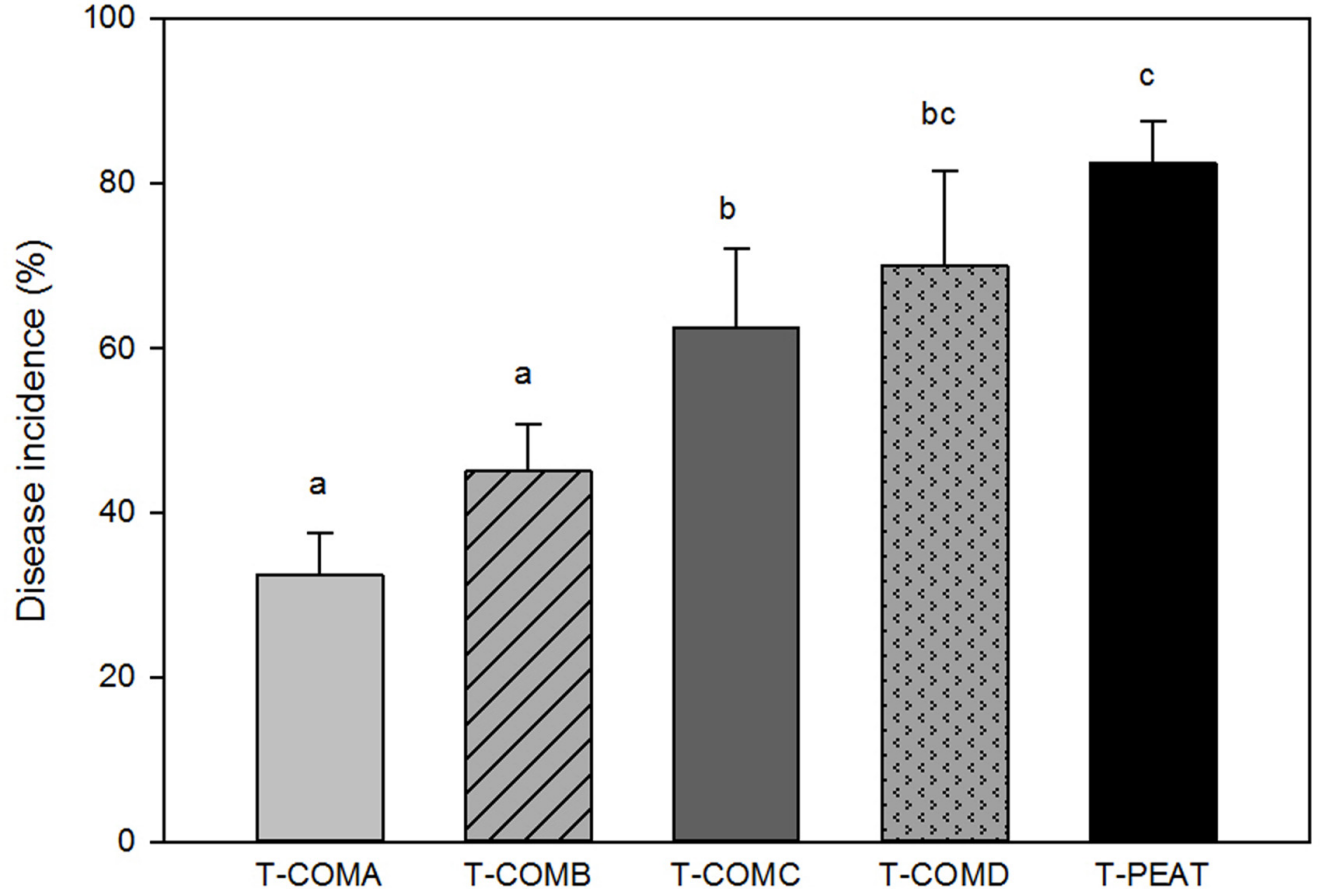
Disease incidence in pepper seedlings artificially inoculated with *P*. *nicotianae*.

The incidence of Phytophthora root rot symptoms of *P*. *nicotianae* on pepper 23 days after inoculation differed significantly among treatments (F = 10.039; *p* = 0.001), indicating that TPEAT treatment (100% peat) was the most conducive growing medium, followed by TCOM-D and TCOM-C, which only reduced the disease incidence by 13% and 23%, respectively, compared to TPEAT (Fig 1). TCOM-A treatment was the most suppressive organic medium against *P*. *nicotianae*, with a reduction of 60% compared to TPEAT, followed by TCOM-B, with a reduction of 50% (Fig 1). Pepper plants in non-infested growth media did not show any symptoms of Phytophthora root rot.

### Composition of the organic fractions in the composts and peat

**Fig 2:**
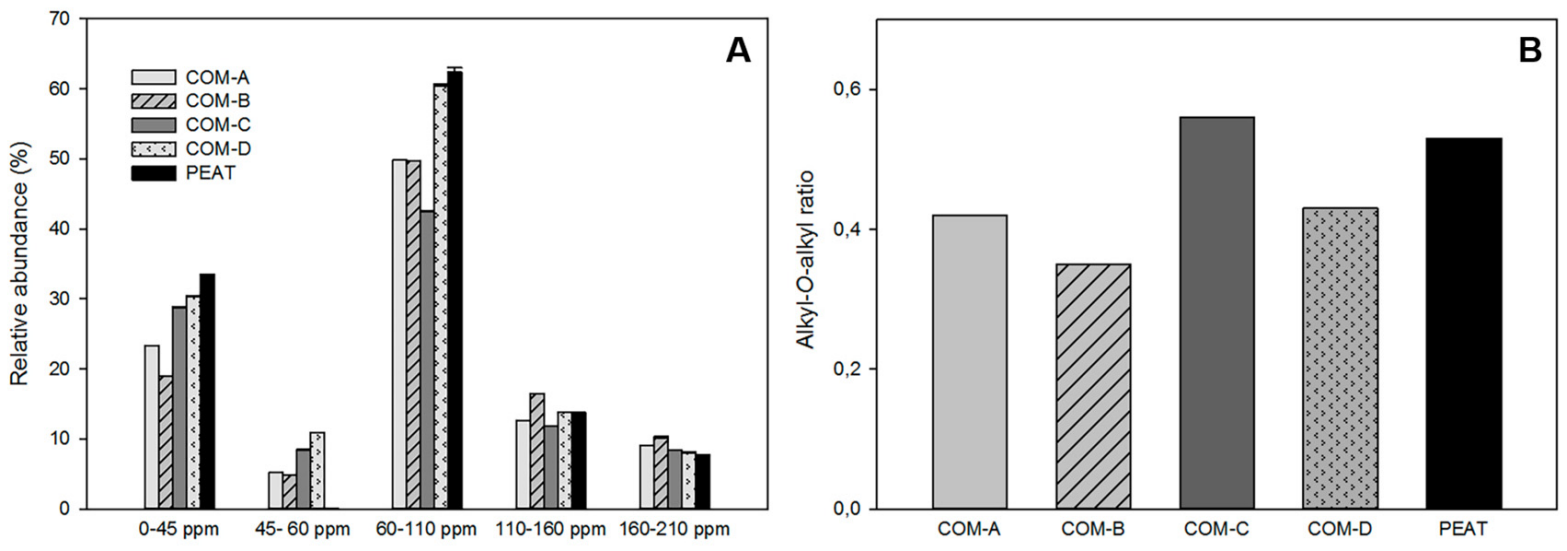
Chemical composition of the composts and peat revealed by ^13^C NMR. (A) Distribution of organic carbon functional groups: 0–45 ppm (aliphatic groups); 45–60 ppm (methoxy groups), 60–110 ppm (carbohydrate groups); 110–160 ppm (aromatic C structures), and 160–210 ppm (carboxyl and ester group). (B) Alkyl/*O*-alkyl ratio values.

The relative integration values for the five specific organic carbon regions from the composts and peat are shown in Fig 2A. Significant differences were observed among fractions (F = 265.22; *p*<0.05). The fraction 0–45 ppm, corresponding to the aliphatic fraction ascribed to lipids, waxes, terpenoids, cutins, and suberins, and the fraction 60–110 ppm, corresponding to the carbohydrate region (polysaccharides, amino acids, amino sugars, lignin substitutes, and others)(18) showed higher relative abundances than the rest of the fractions, namely 45–60 ppm (methoxy groups), 110–160 ppm (aromatic C structures), and 160–210 ppm (carboxyl and ester group) (Fig 2A).

Composts COM-A and COM-B showed lower relative abundances in the aliphatic structure regions compared to composts COM-C, COM-D and peat (P) (Fig 2A). For carbohydrate structure region, COM-C showed the lowest relative abundance. Peat showed the highest relative abundance in both regions (Fig 2A). The relative abundance in the aromatic C structure region followed the trend: COM-B>COM-D>P>COM-A>COM-C, while for carboxyl and ester groups it was COM-B>COM-A>COM-C>COM-D>P (Fig 2A). The alkyl/*O*-alkyl ratio followed the trend COM-C>P>COM-A = COM-D>COM-B (F = 320; *p<0*.*05*) (Fig 2B).

### Metabolomes of the different composts and peat

**Fig 3:**
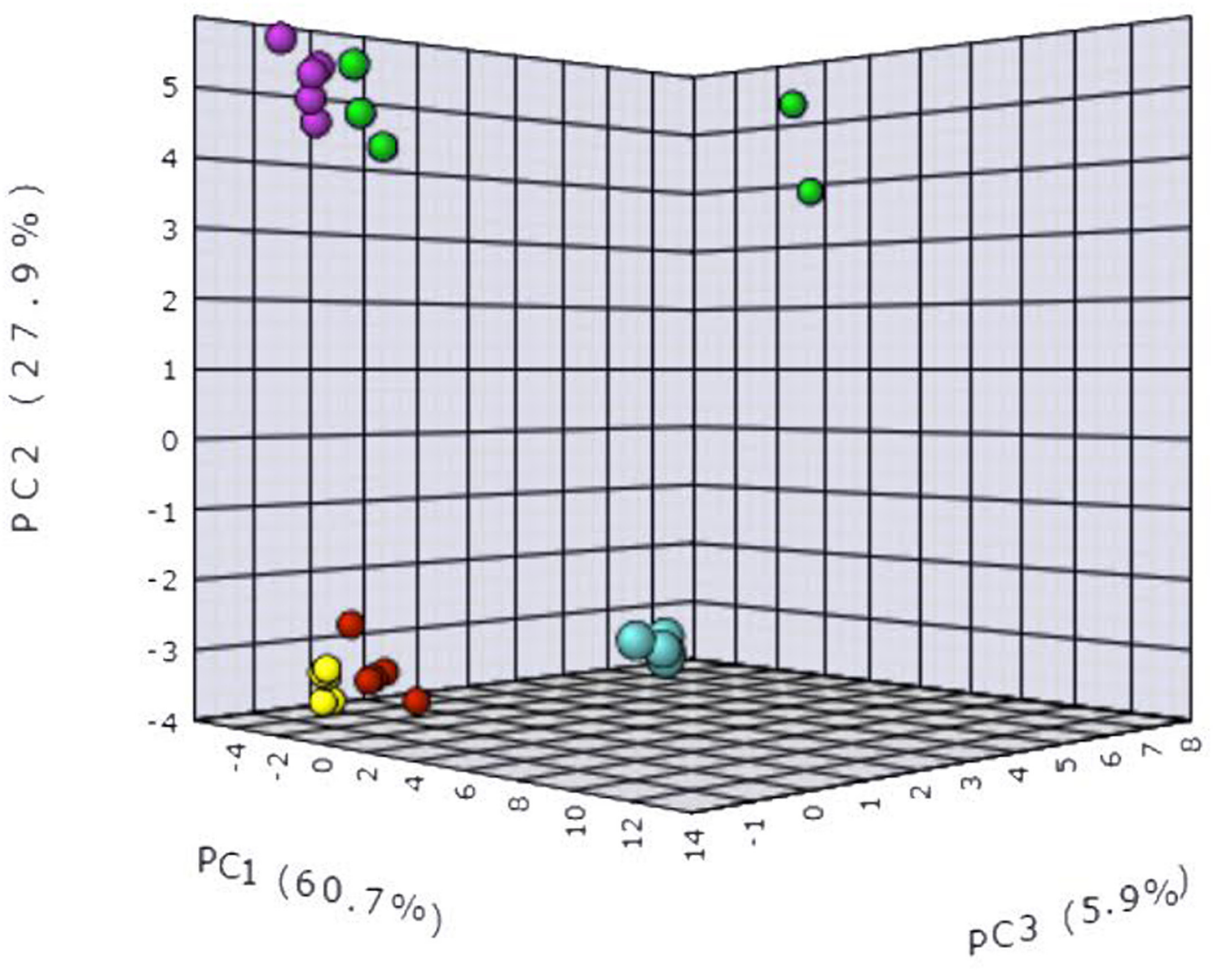
Principal component analysis according to the metabolome obtained from the composts COM-A (green balls), COM-B (pink balls), COM-C (red balls), COM-D (yellow balls), and peat (blue balls), n = 6.

**Fig 4:**
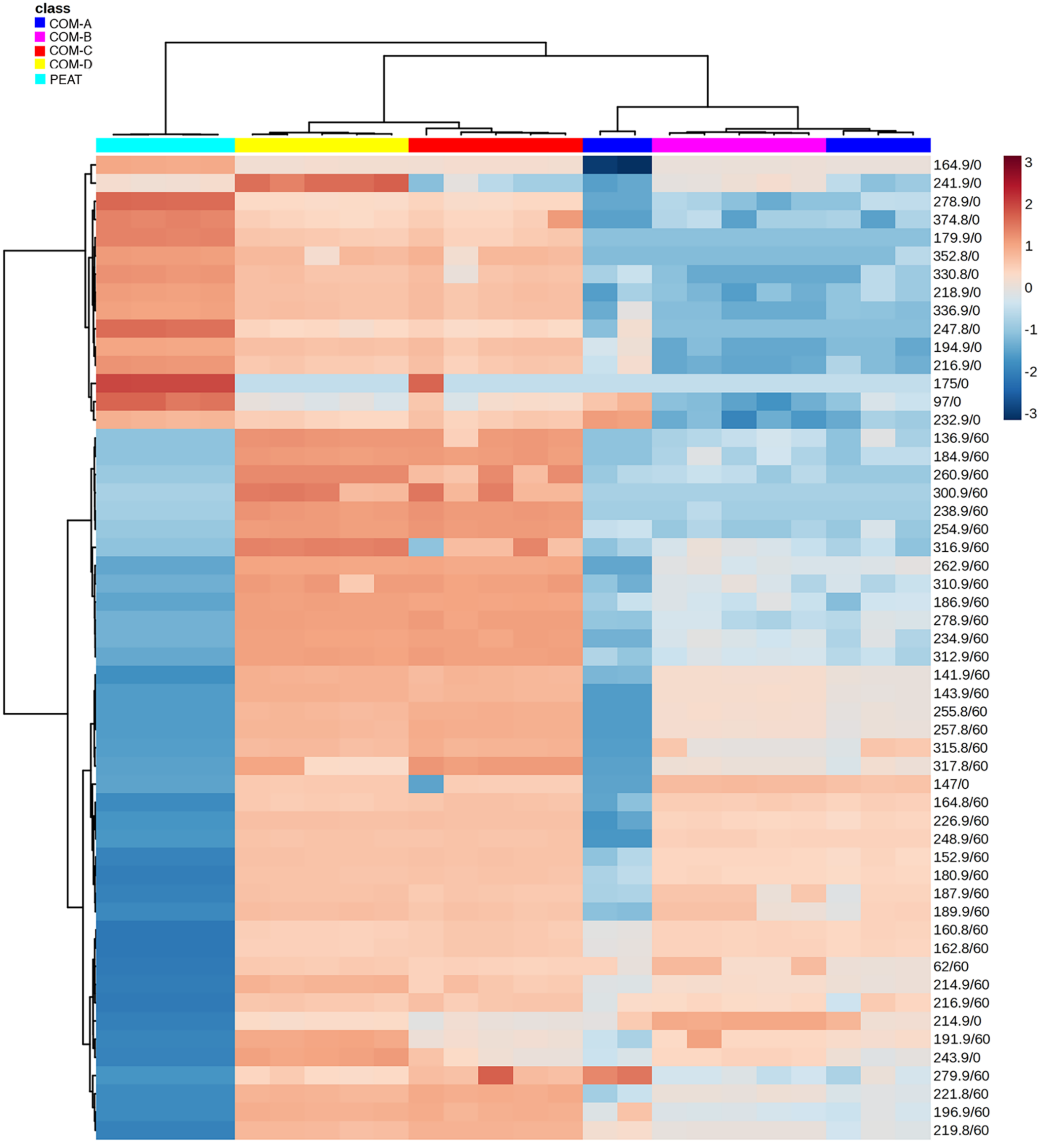
A heat map illustrating the 54 metabolites that differ among the composts and peat. Colors indicate relative quantity of each metabolite.

To find differences among the metabolomes of composts and peat, a principal component analysis (PCA) was applied to construct and validate a statistical model. The two relevant axes explained 88.6% of the variance (PC1 60.7% and PC2 27.9%) (Fig 3). According to Factor 1, multivariate analysis showed three different clusters—peat being separated from one cluster consisting of composts COM-A and COM-B and from another composed of composts COM-C and COM-D (Fig 3). The heat map generated with the mass of the most frequently metabolites found across the profiles showed that most of them were found in lower relative abundance in suppressive composts (Fig 4). Several mass compounds received a high loading score in Factor 1 and contributed the most to the separation of peat from the composts (175; 97; 247.8; 278.9; 179.9; 216.9; 374.8; 232.9; 330.8; 194.9; 336.9; 352.8; 218.9; 164.9) (Fig 4). Other mass compounds (184.9; 260.9; 300.9; 238.9; 254.9; 316.9; 262.9; 310.9; 186.9; 278.9; 234.9; 312.9) contributed to the separation of suppressive from conductive composts.

### Fungal and bacterial communities of the different composts and peat

Of 1904691 reads, we obtained a total of 679687 reads after quality filtering and chimeras, 508043 from 16S rRNA and 171644 from fungal ITS gene sequences across all samples. The OTU clustering and taxonomic assignment, performed using these sequences, yielded 25071 and 3600 individual OTUs from 16S rRNA and fungal ITS genes, respectively.

#### Fungal community composition

**Fig 5:**
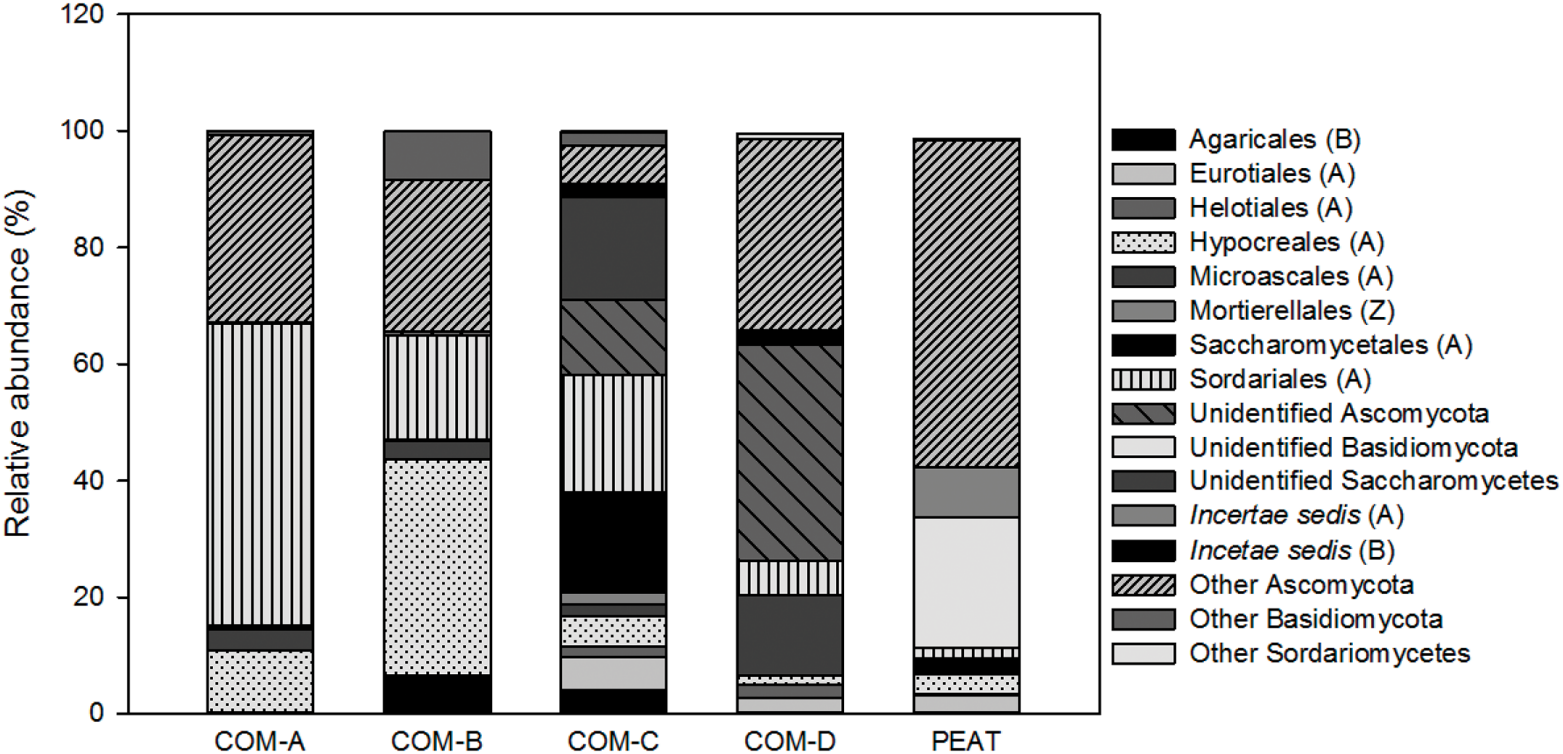
Relative abundances of the fungal orders identified in the composts and peat.

The classified sequences for the composts and peat were affiliated to three fungal phyla. The most abundant phylum was Ascomycota, accounting for 54% of all sequence reads, followed by Basidiomycota (2.34%) and Zygomycota (0.06%). The percentage of sequences classified as other fungi was 6.8%, whereas 36.57% was assigned to unidentified fungi. Examination of the taxonomic structure at the order level (Fig 5) showed that, within the phylum Ascomycota, the most abundant orders were Sordariales, Hypocreales, and Microascales. Composts COM-A and COM-B showed higher relative abundances of Ascomycota (63.14 and 67.38%, respectively), in particular, COM-A had the highest relative abundance of Sordariales and COM-B the highest abundance of Hypocreales (Fig 5). On the other hand, compost COM-C showed a high abundance of Saccharomycetales and compost COM-D of Microascales, while these orders were almost inexistent in peat (Fig 5). Peat showed a high relative abundance of Ascomycota, followed by Basidiomycota (Fig 5). Fungal diversity was observed to be between 2.38–3.75 in composts and 2.64 in peat.

At the genus level, the most abundant classified genera (>1%) for each compost and peat are shown in Table 2. The genera with the highest relative abundances in the composts were *Zopfiella*, *Fusarium*, *Haematonectria*, *Galactomyces*, *Doratomyces*, *Geomyces*, *Coprinellus*, and *Thermomyces*.

**Table 2:** Most abundant fungal genera identified (>1% relative abundance) in the composts and peat.

Phylum	Genus	COM-A	COM-B	COM-C	COM-D	Peat
Ascomycota	*Aspergillus*	0.02	0.01	1.12	0.73	0.00
Ascomycota	*Candida*	0.00	0.00	0.23	0.01	2.72
Ascomycota	*Cephalotheca*	0.00	0.00	0.00	0.76	1.50
Ascomycota	*Chaetomium*	0.28	0.44	1.58	0.03	0.00
Ascomycota	*Doratomyces*	1.29	0.09	0.16	2.64	0.02
Ascomycota	*Fusarium*	4.40	20.2	3.32	0.30	0.00
Ascomycota	*Galactomyces*	0.75	0.24	12.81	0.00	0.00
Ascomycota	*Geomyces*	0.00	0.00	0.00	0.03	8.64
Ascomycota	*Haematonectria*	1.68	9.12	0.44	0.05	0.00
Ascomycota	Hypocrea	0.00	0.00	0.02	0.02	1.83
Ascomycota	*Penicillium*	0.01	0.01	0.02	0.54	2.16
Ascomycota	*Pichia*	0.00	0.01	3.37	0.00	0.00
Ascomycota	*Pseudallescheria*	0.00	0.00	0.18	9.87	0.00
Ascomycota	*Scedosporium*	0.06	2.42	1.35	0.71	0.00
Ascomycota	*Scytalidium*	0.00	0.00	1.88	2.30	0.00
Ascomycota	*Thermomyces*	0.08	0.01	4.38	1.10	0.00
Ascomycota	*Zopfiella*	14.7	0.16	8.87	0.01	0.06
Basidiomycota	*Coprinellus*	0.01	5.41	1.97	0.00	0.00
Basidiomycota	*Myriococcum*	0.09	0.00	2.22	2.62	0.00

#### Bacterial community composition

**Fig 6:**
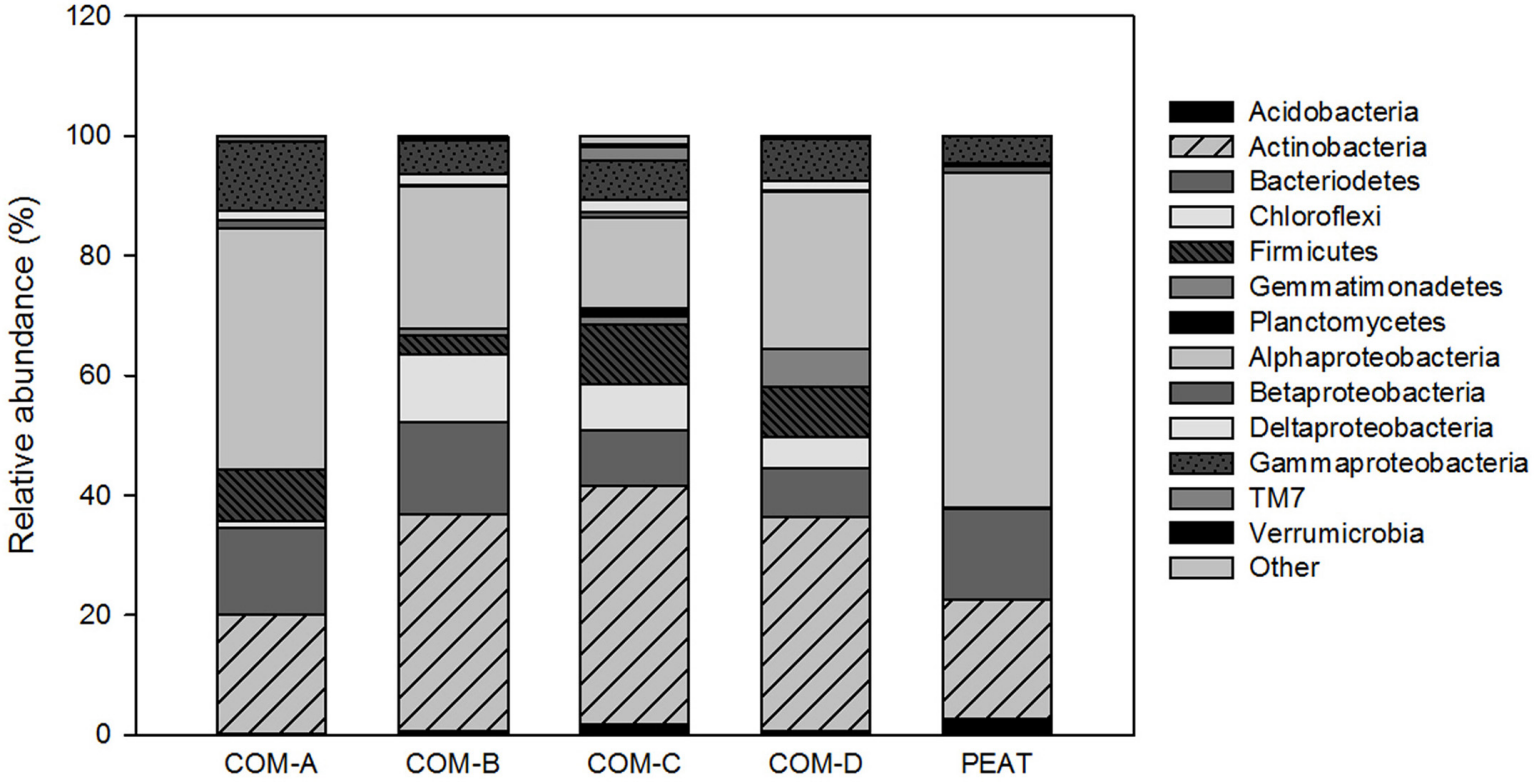
Relative abundances of the bacterial phyla and sub-phyla identified in the composts and peat.

The classified sequences were affiliated with 19 bacterial phyla, and the remaining ones were unassigned. The dominant phyla, found in all composts and peat, were the Proteobacteria (39.89% of total sequence reads), Actinobacteria (30.53%), Bacteroidetes (12.97%), Chloroflexi (6.25%), and—to a lesser extent—Firmicutes (4.87%), Gemmatimonadetes (1.97%), Acidobacteria (1.07%), and TM7 and TM6 (<0.41%). Composts COM-A showed higher relative abundance of Proteobacteria, mainly due to the high abundance of Alphaproteobacteria and Gammaproteobacteria, as well as the lower abundance of Actinobacteria; while COM-B showed higher relative abundance of Chloroflexi (Fig 6). Higher relative abundance of Bacteriodetes were found in compost COM-A and COM-B compared to the rest of compost as was found for Gemmatinomidetes in compost COM-D (Fig 6). Bacterial diversity was observed to be between 6.40–6.70 in composts and 5.5 in peat.

The most abundant classified genera (>1%) for each compost and peat are shown in Table 3. The most frequent genera include: *Microbacterium*, *Mycobacterium*, *Streptomyces*, *Devosia*, and *Rhodoplanes*.

**Table 3:** Most abundant bacteria genera identified (>1% relative abundance) in the composts and peat.

Class	Order	Family	Genus	COM-A	COM-B	COM-C	COM-D	PEAT
Actinobacteria	Actinomycetales	Microbacteriaceae	*Agrococcus*	1.10	1.08	0.23	0.20	0.00
Actinobacteria	Actinomycetales	Microbacteriaceae	*Microbacterium*	3.81	2.72	2.23	0.15	0.03
Actinobacteria	Actinomycetales	Mycobacteriaceae	*Mycobacterium*	0.53	0.51	0.40	0.48	2.86
Actinobacteria	Actinomycetales	Streptomycetaceae	*Streptomyces*	3.63	5.11	2.23	2.72	6.38
Actinobacteria	Actinomycetales	Streptosporangiaceae	*Nonomuraea*	0.05	0.27	1.15	3.02	0.01
Actinobacteria	Actinomycetales	Thermomonosporaceae	*Actinomadura*	0.06	0.38	0.93	1.70	0.84
Flavobacteriia	Flavobacteriales	Flavobacteriaceae	*Arenibacter*	0.00	0.11	0.00	1.75	0.00
Sphingobacteriia	Sphingobacteriales	Sphingobacteriaceae	*Olivibacter*	1.17	0.13	0.00	0.00	0.00
Sphingobacteriia	Sphingobacteriales	Sphingobacteriaceae	*Sphingobacterium*	1.05	0.11	0.02	0.00	0.00
Bacilli	Bacillales	Bacillaceae	*Bacillus*	1.87	0.58	1.45	0.56	0.00
Bacilli	Bacillales	Planococcaceae	*Ureibacillus*	1.62	0.20	0.84	0.74	0.00
Alphaproteobacteria	Rhizobiales	Hyphomicrobiaceae	*Devosia*	4.43	0.63	0.62	2.28	1.04
Alphaproteobacteria	Rhizobiales	Hyphomicrobiaceae	*Hyphomicrobium*	1.19	0.41	1.20	1.75	0.04
Alphaproteobacteria	Rhizobiales	Hyphomicrobiaceae	*Pedomicrobium*	1.86	1.25	0.92	0.90	0.02
Alphaproteobacteria	Rhizobiales	Hyphomicrobiaceae	*Rhodoplanes*	2.51	1.97	1.66	4.16	5.83
Alphaproteobacteria	Rhizobiales	Phyllobacteriaceae	*Mesorhizobium*	2.30	1.19	0.32	2.17	0.62
Alphaproteobacteria	Rhizobiales	Rhizobiaceae	*Agrobacterium*	1.74	0.07	0.01	0.03	0.02
Gammaproteobacteria	Xanthomonadales	Xanthomonadaceae	*Dokdonella*	0.10	0.03	0.04	0.21	2.52
Gammaproteobacteria	Xanthomonadales	Xanthomonadaceae	*Luteimonas*	1.94	0.44	0.19	0.04	0.00

## Discussion

It is essential to understand the community composition of the compost microbiota, not only to gain a better understand of its biology, but also to determine the microorganisms that may be involved in compost suppressiveness. To our knowledge, this is the first study that has used metagenomics methods to characterize the microbiota involved in the control of *P*. *nicotianae*. We found large numbers of sequences for Proteobacteria, Bacteriodetes and Ascomycota in suppressive composts compared to the other composts and peat. Specifically, the Ascomycota phylum was negatively correlated (*r* = -0.953; *p* = 0.012) with Phytophthora root rot incidence. In previous studies, fungal populations have been reported as the main contributors to the biological suppressiveness of compost [12]. These populations become predominant during the composting maturation phase while bacteria populations decrease due to the reduction of substrate quality [33,34]. Moreover, the incorporation of vineyard pruning wastes at different rates into our composts (38–75%) may have led to the development of fungi associated with hardwood compost, as was previously reported by Neher et al. [19]. Within Ascomycota phylum, Sordariales and Hypocreales were identified as the most abundant taxa associated with the suppressive composts, COM-A and COM-B, respectively. One of the most abundant genera in COM-B was *Fusarium*. This genera includes non-pathogenic isolates of *F*. *oxysporum* identified previously as biocontrol agents [35]. On the other hand, in the case of compost COM-A, one of the most abundant genera was *Zopfiella*, which has been reported to produce metabolites active against several species such as *Botrytis cinerea*, *Phytophthora infestans*, or *Pythium ultimum* [36,37]. Although in a much lower relative abundance, both *Fusarium* and *Zopfiella* were found on compost COM-C, which could explain its lower capability to suppress Phytophthora root rot in comparison to peat. By contrast, compost COM-D showed a very low relative abundance of these fungi, while *Pseudallescheria* was the most represented genus. Within this genus, *P*. *boydii* is the most well-known species, since it is a fungal human pathogen that is widespread in soils and produces a fungistatic substance strongly inhibitory to phytopathogens [38].

The fungal community of peat was characterized by the presence of *Geomyces*, a genus of filamentous fungi in the family Myxotrichaceae, known to be phychrophilic and often the most common fungal group found in cold and low-nutrient environments [39]. Relatively high abundances of *Penicillium* and *Hypocrea* were also observed in peat. In spite of the presence of these latter microbes which have been shown to control soil-borne plant pathogens [40,41], peat was conducive to Phytophthora root rot. Similar results have been reported before, the presence of these microorganisms being related to non-suppressive soils [42].

The most suppressive composts (COM-A and COM-B) contained greater relative abundance of Bacteriodetes in comparison with the rest of composts. Indeed, COM-A showed up to double the relative abundance of Proteobacteria (mainly due to Alpha and Gammaproteobacteria) and COM-B showed up to 1.5-times the relative abundance of Chloroflexi, compared to the rest of composts. Conducive composts, COM-C and COM-D, contained relatively more Actinobacteria and Gemmatimonadetes, respectively. Although the positive impact of Actinobacteria on plant disease suppression has been well documented due to their ability to produce a wide array of antibiotics [43], in a recent review of Bonanomi et al. [11], it was concluded that Actinobacteria were only directly correlated with disease suppression in a limited number of experimental cases. Although the presence of some bacteria (Gamma-proteobacteria, Firmicutes, and Actinobacteria) has been used as an indicator of disease suppression [42], no positive correlations were found among Phytophthora root rot control and bacteria populations in the present study.

The metagenomics analysis showed that compost microbial communities are vast and diverse, maintaining a high degree of uniqueness according to the nature of compost. It is not surprising that compost microbiota was highly affected by the chemical heterogeneity of the substrates. The concentration and availability of nutrients within organic matter play a critical role in regulating and maintaining microbial populations. During the composting process, once the less recalcitrant components (for example, oligosaccharides, organic acids, hemicellulose and cellulose) are rapidly degraded by the microbial activity, the remaining highly recalcitrant compounds (for example, lignin or the cellulose encrusted in lignin) promote the presence of microorganisms which are able to degrade them [34,44,45]. In this respect, the higher amount of vineyard pruning wastes used in compost COM-A and COM-B (75 and 67%, respectively), may have promoted high microbial activity as was demonstrated by the high levels of dehydrogenase activity. This microbial parameter has been widely used as an indicator of overall soil microbial activity [46–48]. Higher OM level, in fact, can provide enough substrate to support higher microbial biomass and as a consequence higher enzyme production [49]. Hoitink et al. [50] proposed that the concentration of cellulose and lignin in composted manure define the longevity of suppressive effect. The high levels of recalcitrant compounds of vineyard pruning waste may favor the proliferation of microorganisms with the ability to degrade the plant cell wall through a set of synergistically enzymes [42]. As a result, soluble compounds may be released and used by other microorganisms inducing shifts in the composition of a large microbial community, leading to a “general suppression phenomenon” [8,13,51]. *Phytophthora* spp. is often considered highly sensitive to microbial competition since it depends on exogenous carbon sources for spore germination to infect host plants [10,52]. As a matter of fact, in previous studies a direct relationship between compost microbial activity and suppression of Pythium and Phytophthora root rots has been reported [7,50]. Moreover, it has been shown the quality of OM somehow affects the permanence of suppressive effects [13,50,53].

To understand in detail the chemical characteristics of the composts used in the present study and the influence in their suppressive capabilities, ^13^C-CPMAS-NMR spectroscopy was used to analyze their organic matter fractions [15,29]. Boehm et al. [54], using this technique, demonstrated that peat suppressiveness to *P*. *ultimum* decreases because of the progressive depletion of carbohydrates and easily degradable organic compounds. Conversely, our study observed a positive correlation (*r* = 0.519; *p*<0.05) between relative abundance of carbohydrates (60–110 ppm fraction) and Phytophthora root rot incidence. As proposed by Castaño et al. [29], high content of lignin-like structures (e.g. in compost COM-A and COM-B) may mask values in the carbohydrates region, hindering a possible positive correlation between relative abundance of this region and a possible suppressive effect. These results suggest that not only the content but also the bio-availability of cellulose play an important role regarding organic matter suppressiveness [29]. Thus, the bio-availability of carbohydrates should be considered crucial for suppression of *Phytophthora* spp., as has been previously considered for other pathogens such as *Pythium* spp. [13] and *Rhizoctonia solani* [55].

Peat and compost COM-D treatments showed the highest disease incidence and the lowest microbial activity, but surprisingly, also showed the highest levels of 60–110 ppm profile. These controversial results may be explained assuming that in peat and COM-D the cellulose is enclosed in lignin and is not available for microorganisms; as a consequence these media are not capable of sustaining high microbial activity. Furthermore, some of these carbohydrates may be released upon the death of microbes [29]. This is in concordance with the high levels of Alkyl-*O*-alkyl ratio found in peat, which could be interpreted as the result of a progressive degradation of carbohydrates. We used this ratio as an index of humification and stabilization of the organic matter as suggested by other authors [44]. Also, peat is characterized by a low bacterial diversity in comparison with composts. In spite of the high content (40–42% of lignin) of compounds recalcitrant to biodegradation in peat, the low amount or absence of microorganisms which are able to degrade these complex compounds, could lead to an environment not conducive to the microbial activity associated with soil suppressiveness.

Along with peat, COM-C presented the highest levels of Alkyl-*O*-alkyl ratio, which could be explained by the low levels of polysaccharides found in this compost. Its composition was based on lower content of vineyard pruning waste (38%) and high sludge content (20%). According to Tittarelli et al. [56], the sludge produces a high mineralisation rate during composting as the result of incorporating a high amount of easily-mineralisable carbon and mineral components. COM-C is an excessively stabilized compost, which is unable to sustain a high microbial activity (as indicated by the low dehydrogenase activity levels) and therefore, it is characterized by a low ability to supress disease.

In contrast with Lopez-Gonzalez et al. [34], we believe that it is useful to determine the identity of microorganisms in different composts, if their presence is involved in the suppressive capability of a compost. In the present study, in spite of the differences found in compost microbial composition, we found a correlation between suppressiveness to Phytophthora root rot and the level of microbial activity, whereas we did not find any correlation between disease suppressiveness and a single microbial taxon. By contrast, in a recent study, Yu et al. [22] suggested a potential role of the bacteria Acidobacteria Gp14 and Cystobasidiomycetes fungi in the suppression of Pythium wilt disease. Microbial diversity (Shannon diversity) did not correlate with disease suppression, although higher levels of bacterial diversity were observed in COM-A and COM-B.

Metagenomics has proved to be a powerful approach to explore microbial communities in composts. However, due to the inability of DNA-based molecular techniques to provide information of the gene expression (functionality) as it occurs under *in situ* conditions [57], the use of postgenomic approaches such as metametabolomics has been suggested [58]. However, there are few metabolic studies on soils [59–62] and, to our knowledge, this is the first one on composts. Metabolomics attempts to capture the complexity of metabolic networks via the comprehensive characterization of the small-molecule metabolites (e.g. amino acids, sugars, and lipids) in biological systems [24]. Thanks to its sensitivity, this approach has a high potential to elucidate changes in the levels of soil metabolites. PCA demonstrated that the extracts from the composts and peat clustered in a manner that was correlated with their ability to suppress Phytophthora root rot. Similarly, Rochford et al. [60] observed that growth inhibition against *Bacillus subtilis* of different soil extracts was strongly correlated with their metabolic profile. Metabolite composition is governed by the extant microbial communities in the substrate and it is well-known that pathogen inhibition may be mediated by the secretion of antibiotics or antimicrobial compounds [63,64]. For instance, over two-thirds of all natural antibiotics are derived from *Streptomyces* spp. [63]. Some species within this genus can produce antifungal compounds such as tuberdicidin, phosphalactomycin, and candicidin [65–67]. Also, the antibiotics zwittermicin A and kanosamine produced by the biological control agent *Bacillus cereus* UW85 is active against *Phytophthora* spp. [68,69]. It is important to underline that some bacterial strains, which are not biological control agents by themselves, can act synergically as part of microbial consortia [70].

The metabolic approach employed here was based on water extraction followed by data acquisition by liquid chromatrography-mass spectrophotometry-HPLC-MS; although it proved to be very effective in highlighting the diversity of compost microbiome, results cannot be directly compared with previous studies, where different extraction methods were used [60,71]. Results we obtained are to be considered preliminary and the involvement of metabolites in the ability of composts to suppress *P*. *nicotianae* deserves further investigation.

It is important to note that compost contributes to disease suppression in a complex manner which makes it difficult to establish direct correlation among the analyzed parameters and suppressiveness. Nevertheless, future research can build on the results present here to determine which materials are the best to achieve the desired goals of disease suppression.

## Conclusions

Although all composts contained abundant and diverse microbial communities, not all of them were able to control Phytophthora root rot of pepper plants to the same extent. These differences seem to be related to the different composition of microbiome, which in turn was correlated with the nature of materials used. The most suppressive composts, COM-A and COM-B, were made of a relatively higher amount of vineyard pruning waste and showed a higher level of total microbial activity. It can be hypothesized that the suppressiveness against *P*. *nicotianae* may be driven by the availability of carbohydrates derived from the original materials and their ability to sustain a high microbial activity. The relative abundance of the Ascomycota phylum, mainly of the orders Sordariales and Hypocreales, was correlated with the compost suppressiveness; while in the case of bacterial populations, no correlations were found with suppressiveness. The metametabolic analysis of composts and peat demonstrated the relevance of metabolites in the ability of composts to control *P*. *nicotianae*. The conjugation of different techniques, including omics approaches (metagenomics and metametabolomics), to characterize composts, proved its usefulness in clarifying the complex structure of microbial communities in composts and its role in suppressiveness.
